# The human DEPhOsphorylation Database DEPOD: 2019 update

**DOI:** 10.1093/database/baz133

**Published:** 2019-12-14

**Authors:** Nikhil P Damle, Maja Köhn

**Affiliations:** Signalling Research Centres BIOSS and CIBSS, Faculty of Biology, University of Freiburg, Schänzlestrasse 18, 79104 Freiburg, Germany

## Abstract

The human **Dep**h**o**sphorylation **D**atabase (DEPOD) is a manually curated resource that harbors human phosphatases, their protein and non-protein substrates, dephosphorylation sites and the associated signaling pathways. We report here an update to DEPOD by integrating and/or linking to annotations from 69 other open access databases including disease associations, phosphorylating kinases, protein interactions, and also genome browsers. We also provide tools to visualize protein interactions, protein structures, phosphorylation networks, evolutionary conservation of proteins, dephosphorylation sites, and short linear motifs within various proteins. The updated version of DEPOD contains 254 human phosphatases, 336 protein and 83 non-protein substrates, and 1215 manually curated phosphatase-substrate relationships. In addition, we have improved the data access as all the data in DEPOD can now be easily downloaded in a user-friendly format. With multiple significant improvements, DEPOD continues serving as a key resource for research on phosphatase-kinase networks.

**Database URL**: www.depod.org

## Introduction

Phosphorylation is a reversible post-translational modification. Knowledge about protein kinases and phosphatases is critical to understand the phosphoregulation of a signaling event comprehensively. Advances in experimental and computational approaches led to the development of several kinase-centric resources and tools, whereas phosphatase-centric resources are very few. The human **DEP**h**O**sphorylation **D**atabase (DEPOD) was the first resource that curated information from literature about the phosphatase genes in humans along with their protein and non-protein substrates, the dephosphorylation sites, the protein interactions involving them and the signaling pathways regulated by them (1,2). Currently available other phosphatase-related resources either do not provide information about their substrates (PTP-central (3), Phosphatome. Net (4), EKPD (5) and iEKPD (6)) or provide information only about the protein substrates (HuPho) (7). Additionally, many of them rely on DEPOD for information on the phosphatase genes in humans and their substrates, and have not been updated in years, and therefore do not reflect the current knowledge on phosphatases and their substrates.

We have now updated and also improved DEPOD significantly on the following three fronts: A. compilation, by adding new phosphatases and substrates; B. annotations, by integrating annotations from 69 other open access databases, and including two new modules on disease associations and protein kinases that are experimentally known to phosphorylate a given protein in DEPOD; and C. data access and visualization, by better organizing and interlinking the information and incorporating new visualization tools to facilitate data navigation.

DEPOD is now a unique resource that gives information about the bidirectional regulation of a phosphorylation event. New hypotheses may now be formulated based on this information and also based on the evolutionary conservation of proteins, the dephosphorylation sites and short linear motifs (SLiMs) within them. Disease associations in DEPOD may be of great value to increasing efforts to target phosphatases therapeutically (8). Overall, DEPOD will be a very valuable resource for the phosphorylation signaling community and also cater to the needs of wider research interests.

## Organization of DEPOD

**Figure 1:**
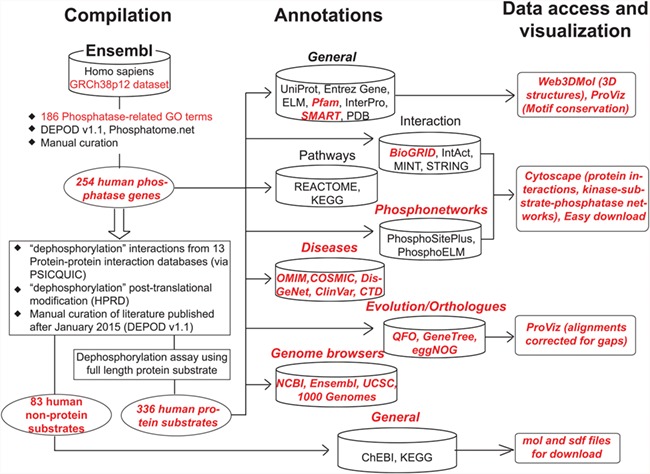
Schematic organization of DEPOD. DEPOD is organized in the following three modules: compilation, annotations and data access and visualization. Details on each are described in text. Red font indicates new additions or improvements in the current version over earlier versions.

Figure 1 shows the overall organization of DEPOD consisting of the following three modules: A. compilation, B. annotations and C. access and visualization of data. Supplementary Figure S1 depicts the entity relationship diagram showing its architecture as a relational database.

### A. Compilation of phosphatase genes and their substrates

Phosphatase genes in humans were fetched from Ensembl (GRCh38p12) using a keyword-based search. Candidates were filtered using 186 phosphatase-related gene ontology (GO) terms (Supplementary Table S1) to filter out non-phosphatase genes from the list. Remaining genes were compared with previous versions (1,2) of DEPOD and also with two other recently developed phosphatase-related resources—Phosphatome. Net (4) and iEKPD (6) to finally compile a list of 254 phosphatase genes in humans. These were then reclassified into superfamilies based on their CATH fold (9) and subsequently into families based on the sequences of their catalytic domains (Supplementary Table S2). This classification was carried out as reported previously (1, 2). Briefly, the catalytic domains of the phosphatases were fetched from their Uniprot FASTA sequence using the Pfam domain definitions such as Y_phosphatase for Tyr-phosphatases and metallophosphatase domain for Ser/Thr-specific phosphatases. In cases where more than one phosphatase domain appeared in a single polypeptide, the domain having the catalytic motif signature as defined by Alonso *et al*. (10) was used for classification.

Unlike in DEPODv1.1, we have now renamed the superfamilies and families so that the nomenclature sufficiently reflects the substrate specificities of phosphatases and relates to the historic classes. For example, ‘family 1’ in DEPODv1.1 is now called ‘Class I Cys-based PTPs’ in accordance with previous literature (4, 11). Phylogenetic trees constructed using neighbor-joining algorithm in MEGA suite (12) and the catalytic domains of the members belonging to each of these superfamilies can be easily accessed using the ‘view tree’ links on the ‘structure-based family’ tab. The tree visualizations have been created using interactive tree of life (iTOL) (13). We now also provide in the download section the comparison between different databases and classifications, to provide clarity to this ongoing research. Of note, DEPOD contains more phosphatases than other recently developed phosphatase-related resources (4, 6), largely due to the inclusion of non-protein phosphatases and manual curation of the entries.

Fourteen different protein interaction databases were queried via PSICQUIC service (14) using the ‘dephosphorylation’ keyword. In-house scripts were developed to merge the outputs and create a list of unique dephosphorylation interactions. Interactions where no literature evidence was available (PubMed ID unassigned) were filtered out. Interactions where only fragments of the proteins (such as a peptide stretch or only a single domain in a multidomain protein) were used during dephosphorylation assay were also excluded. Remaining literature evidences published after January 2015 (DEPODv1.1) or not inspected during the development of DEPODv1.1 were inspected manually to compile a list of protein and non-protein substrates of the aforementioned phosphatase genes. A protein is regarded as a substrate only if the dephosphorylation of the full-length protein has been convincingly established using either or both of the *in vitro* and *in vivo* (in human cell lines) assays by single or multiple research groups. A complete list of PubMed IDs processed during this compilation along with the corresponding decisions and the underlying rationale can be found in Supplementary Table S3. Taken together, DEPOD now holds information about 336 protein substrates and 83 non-protein substrates of human phosphatases representing 1215 dephosphorylation interactions. The reliability scores of these phosphatase–substrate interactions were assigned as described in DEPODv1.1.

### B. New annotations in DEPOD

**Figure 2:**
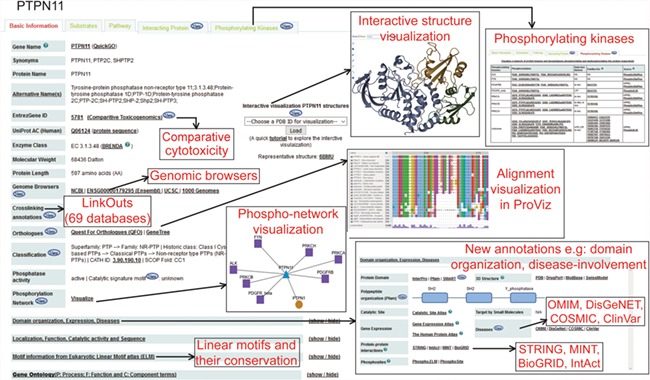
A typical protein entry in DEPOD. Various annotation fields are depicted and newly added annotations are highlighted separately. Phosphatase entries have a ‘Substrates’ tab that harbors information about its protein and non-protein substrates. Substrate entries have a ‘Phosphatases’ tab that includes information about phosphatases that dephosphorylate them.

Figure 2 and Supplementary Figure S2 show a typical protein and a non-protein entry in DEPOD, respectively. DEPOD now provides several new and useful annotations for phosphatases and their substrates.

#### Disease associations.

We have incorporated diseases associated with the phosphatases and their substrates from four different databases, namely COSMIC (15), DisGeNet (16,17), OMIM (https://omim.org/) and ClinVar (18) along with the corresponding reliability scores assigned by the parent databases. (‘Domain organization, Expression, Diseases’ subsection in the ‘Basic Information’ tab of each entry). The link to the PhosphoSitePlus database provided in this section may also be used to gain disease information on the gene/protein of interest and on involved or mutated phosphorylated sites.

#### Domain organization.

Under the same section, we also provide links to the corresponding entry pages in Pfam (19), SMART (20) and InterPro (21) databases. For a quick overview, we now provide a visual schematic domain organization of a polypeptide with links to the corresponding domain families in Pfam.

#### Genomic annotations.

Links to four genomic browsers (NCBI, UCSC, 1000Genomes and Ensembl) have been provided to explore genomic features of an entity. **C**omparative **T**oxicogenomics **D**atabase (CTD) (22) has also been linked to give a glimpse of the chemical–gene interactions and other manually curated information provided there.

#### Crosslinks.

Each protein entry in DEPOD has now been linked with 69 other publicly accessible databases by creating a mapping between DEPOD entry names and other identifiers used in the corresponding databases. Some of these identifiers can be accessed using the links provided in DEPOD entries including the ‘Query our ID-mapping table’ link while others are provided as an excel sheet in the ‘Download’ section.

#### Catalytic signature motifs.

For protein tyrosine phosphatases, DEPOD now provides their signature catalytic motifs incorporated from Alonso *et al*. (10).

#### Protein interactions.

DEPOD provides protein interacting partners of phosphatases and their protein substrates under the ‘Interacting Proteins’ tab along with annotations about the detection methods, interaction types, interaction scores and links to original literature evidences supporting the interactions from BioGRID (23), IntAct (24) and MINT (25). The reliability scores for the protein interactions and the source publications have been directly imported from the corresponding databases.

#### Phosphorylating kinases.

We have added a new module on protein kinases that are experimentally demonstrated to phosphorylate various protein entries in DEPOD by integrating information from PhosphoSitePlus (26), PhosphoELM (27) and HPRD (28). Each phosphorylation site and the five amino acids on its either sides are annotated with the phosphorylation detection method and links to original supporting publications. In addition, evolutionary conservation of each site across metazoan and other clades may be visualized by clicking on each of the sites.

**Figure 3:**
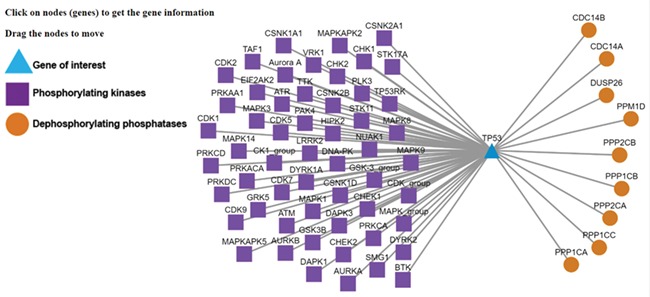
Kinase-substrate-phosphatase relationships (PSKRs) of TP53 in DEPOD. DEPOD imports the information about the protein kinases (purple squares) experimentally demonstrated to phosphorylate a gene (TP53 shown as blue triangle in this case) from PhosphoSitePlus, PhosphoELM and HPRD. The information about phosphatases (orange spheres) that dephosphorylate this gene is manually curated in DEPOD. PSKRs in DEPOD inform about bidirectional phosphoregulation of a gene of interest. At present, DEPOD distinguishes dephosphorylation of a substrate by different isoforms of a phosphatase only in cases where literature evidence specifically exists for that isoform. In the absence of any such evidence, all isoforms of that phosphatase are considered to dephosphorylate a given substrate (here for example PPP2CA and PPP2CB). The figure was created using Cytoscape (45).

#### Evolutionary conservation.

DEPOD provides information about the evolutionary conservation of entire proteins, the SLiMs and also the phospho and dephosphorylation sites within proteins. This information enables users to formulate hypotheses about the functional importance of a particular motif or site depending on whether or not it is conserved.

#### Annotations for non-protein substrates.

Non-protein substrates of phosphatases is a unique feature of DEPOD. Substantially improving this area, non-protein substrates have now been annotated with their molecular formulae, SMILES and InChI notations and InChI keys. They have also been linked to KEGG (29), ChEBI (30), ChEMBL (31), PDB-CCD (32) and PubChem (33). Information about the 69 external databases may be found under the ‘About DEPOD’ section on the left-hand side panel.

### C. Data access and visualization

DEPOD can be queried, accessed and navigated in a number of different ways. All protein entries in DEPOD can be queried using UniProt accessions, gene and protein names, NCBI gene identifiers and GenBank identifiers. Non-protein substrates may be queried using ChEBI or KEGG identifiers, compound names, molecular formulae and SMILES notations. Other query mechanisms such as quicksearch utility, KEGG or REACTOME pathway-based queries, dephosphorylation site-based queries and sequence-based queries have been retained from DEPODv1.1.

Data in DEPOD can now be easily accessed via various links provided with each entry. Users can download FASTA formatted sequences of protein substrates of phosphatases under the ‘Protein substrates’ sub-tab under the ‘Substrates’ tab in each phosphatase entry. The ‘Interacting Proteins’ tab provides a link to download excel sheets containing the protein interacting partners of protein entries in DEPOD from the three protein interaction databases. A non-redundant list created by merging all protein interacting partners together can also be downloaded under the ‘All’ sub-tab. The ‘Basic Information’ tab for non-protein entries provides links to download structures in*.mol* and*.sdf* formats.

The four major ways to navigate DEPOD have been retained from v1.1. Human phosphatases can be browsed using a link on the homepage. CATH (9) fold-based structural classification implemented in DEPOD is provided under the ‘Structure-based family’ tab. We renamed the phosphatase superfamilies to reflect as closely as possible their substrate preferences and to relate to their historical classification. Links associated with each superfamily, family, EC number and the historic class lead to a table describing the members belonging to that group along with their SCOP (34), CATH (9) and EC identifiers (35), DEPOD classification and the catalytic activity status. In addition, FASTA formatted polypeptide sequences of these members can be downloaded using a link provided with the table. We have now also added phylogenetic trees for each superfamily of phosphatases with color codes representing the member families within each of them. Pathways regulated by phosphatases or involving its substrates are listed under the ‘Pathway’ tab. Other phosphatases and substrates also involved in the same pathway can be accessed by clicking on the corresponding pathway identifiers. DEPOD now stores information about 299 KEGG pathways (29) and 876 REACTOME pathways (36) along with the phosphatases and their substrates involved in them.

Under the ‘Basic Information’ tab, users can select and load PDB identifiers of their choice and interactively visualize the corresponding structure using Web3DMol (37). A link on how to use this tool has also been provided for an informed usage. Visualization of evolutionary conservation of the entire protein and the SLiMs and phospho and dephosphorylation sites within proteins is now possible through ProViz tool (38) using simple clickable links. We enabled interactive visualization of protein interaction networks and kinase-substrate-phosphatase relations (Supplementary Figure S3) by incorporating cytoscpe.js libraries (39) with each node linked to its NCBI gene annotations.

### Exploring DEPOD

We now explain the use of some of the new and unique features of the database with TP53 as example, which users can search using the gene symbol or protein name under the ‘Search Substrates’ tab. Lack of phosphorylation at Thr55 of TP53 leads to its nuclear localization and eventually DNA damage-induced cell death (40). Manually curated information about the dephosphorylating phosphatases in DEPOD (PP2A—in the ‘Phosphatases’ tab of the TP53 entry) along with the information about the phosphorylating kinases integrated from PhosphoSitePlus and Phopsho.ELM (GRK5, MAPK1 and TAF1—‘Phosphorylating kinases’ tab) and the neighboring sequence context (integrated from UniProt) will facilitate interpretations about phosphoregulation of this site without the need to visit different databases individually. A mouse click on this site will reveal the conservation of this entire stretch across different model organisms. This information may for example be used to design amino acid substitutions in a certain sequence neighborhood. Clicking on the phosphorylation network visualization link (‘Phosphorylation Network’ section on the ‘Basic Information’ tab or a separate link under the ‘Phosphorylating Kinases’ tab) will also provide information about other kinases and phosphatases that regulate TP53 (Figure 3). This information is valuable to understand alternate regulatory mechanisms.

## Usage and Licensing

All data in DEPOD remains freely accessible and usable for academic and non-profitable usage and is provided under the ‘Download’ section on the homepage. Licensing information for non-academic and for-profit users should be inquired directly under depod@bioss.uni-freiburg.de. More information on how to use and navigate DEPOD along with a brief description of different fields in DEPOD entries can be found under the ‘User Manual’ and ‘About DEPOD’ links in the left-hand navigation panel.

## Concluding Remarks

DEPOD has been used in many studies to formulate hypotheses or as a key resource for phosphatase substrates and networks (e.g. (2, 41, 42). With several significant and useful additions, DEPOD continues to serve as a curated and comprehensive resource for human phosphatases and their substrates. It is unique in harboring both the protein and the non-protein substrates of human phosphatases and similarly both active and inactive human phosphatases. Through annotations on protein kinases that phosphorylate human phosphatases and their substrates, DEPOD informs users about the bidirectional phosphoregulation of a signaling event. This knowledge may help in understanding individual signaling events comprehensively and also the signaling networks in general as specific targeted experiments can be designed based on it. This knowledge may also be useful in clinical studies. Phosphatases are widely pursued as potential drug targets (8). Owing to the conserved active sites within one phosphatase family, inhibition of a particular phosphatase may have several undesired effects (43,44). DEPOD now provides knowledge about the diseases associated with human phosphatases and their substrates, which might help in proposing hypotheses about other potential signaling pathways that might also be inhibited by the drug candidates. This is key piece of information in designing selective inhibitors of phosphatases (44). Information about the extent of conservation of a particular motif or a dephosphorylation site provided by DEPOD may be useful in investigating its functional role in cellular signaling and may also be helpful in designing activity modulators of phosphatases, widely used tools to investigate phosphatase-signaling networks. In addition to encouraging the development of other phosphatase-centric resources, DEPOD also highlights the need to identify more substrates of phosphatases in humans.

## Supplementary Material

DEPOD_supplementary_data_r1_baz133

DEPOD_Supplementary_Table_S1_baz133

DEPOD_Supplementary_Table_S2_R1_baz133

DEPOD_Supplementary_Table_S3_baz133
