# Supplementary material for: The human DEPhOsphorylation Database DEPOD: 2019 update
Source: Database (Oxford). 2019 Dec 14;2019:baz133. doi: 10.1093/database/baz133 (PMC6911163; doi:10.1093/database/baz133)
Supplement: DEPOD_supplementary_data_r1_baz133 [file depod_supplementary_data_r1_baz133.pdf]

Supplementary Data to:

The human DEPhOsphorylation Database DEPOD: 2019 update

Nikhil P. Damle[1], Maja Köhn[1,*]

[1]Signalling Research Centres BIOSS and CIBSS, Faculty of Biology, University of Freiburg, Schänzlestrasse 18, 79104 Freiburg, Germany

* Correspondence: Email: maja.koehn@bioss.uni-freiburg.de

SUPPLEMENTARY FIGURES

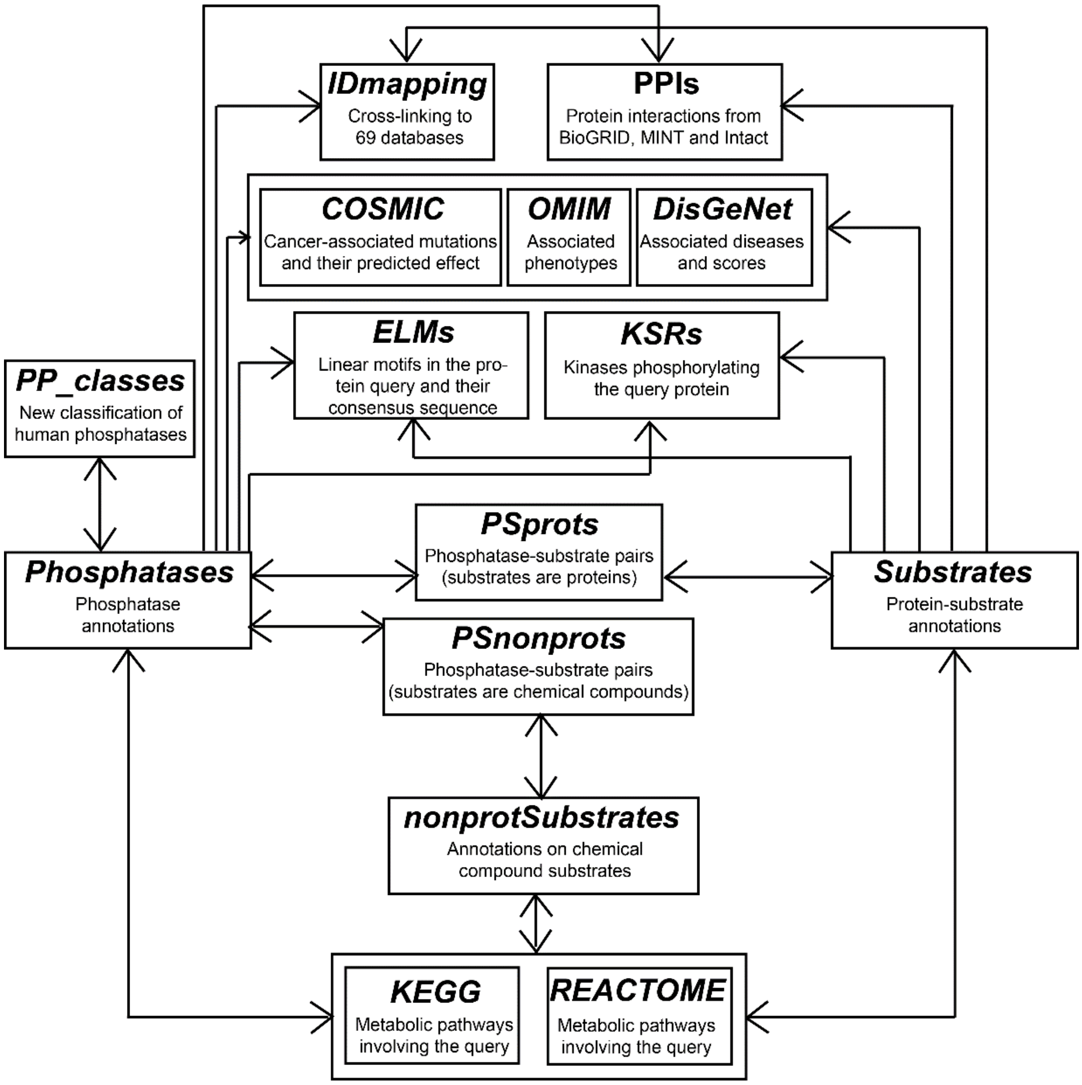

Supplementary Figure S1: Entity relationship (ER) diagram for DEPOD. This ER diagram depicts various core tables stored in DEPOD with a brief description of each. Arrow directions indicate interlinking and query possibilities among different tables facilitated via the web interface.

CHEBI: 16905

Basic Information | Phosphatases | Pathway

**Name**	keto-D-fructose 1,6-bisphosphate
**Synonyms**	01,6-di-o-phosphono-d-fructose;<br>D-fructose 1,6-bisphosphate;<br>D-fructose 1,6-bis(dihydrogen phosphate);
**Definition**	A ketohexose bisphosphate that is D-fructose substituted by phosphate groups at positions 1 and 6.
**Molecular Weight (Exact mass)**	340.1157 (339.996)
**Molecular Formula** New	C6H14O12P2
**SMILES** New	[H][C@@](O)(COP(O)(O)=O)[C@@]([H])(O)[C@]([H])(O)C(=O)COP(O)(O)=O
**InChI** New	InChI=1S/C6H14O12P2/c7-3(1-17-19(11,12)13)5(9)6(10)4(8)2-18-20(14,15)16/h3,5-7,9-10H,1-2H2,(H2,11,12,13)(H2,14,15,16)/t3-,5-,6-/m1/s1
**InChI Key** New	XPYBSIWDXQFNMH-UYFOZJQFSA-N
**Crosslinking annotations** New	**KEGG:C00354** | 3DMET:B04673 | CAS:488-69-7 | **ChEBI:16905** | **ChEBI:37736** | **ChEMBL:CHEMBL1089962** | **ChEMBL:CHEMBL2111113** | **ChEMBL:CHEMBL97893** | **KNApSAcK:C00007386** | NIKKAJI:J15.941G | **PDB-CCD:AFP** | **PDB-CCD:FBP** | **PubChem:3647**

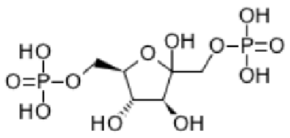

C00354

New Download: **mol** | **sdf**

Supplementary Figure S2: A typical non-protein substrate entry in DEPOD. Several new annotations such as molecular formula, SMILES and InChI notations and InChI keys have been added. Cross-linking annotations to several small-molecule databases have been provided. Structures can also be downloaded in *.mol* and *.sdf* formats.

SUPPLEMENTARY TABLE CAPTIONS

Supplementary Table S1: 186 GO terms with which human phosphatases have been compiled.

Supplementary Table S2: Human phosphatase genes in DEPOD and a comparison between their classification into Superfamilies, Familes and Subfamilies by DEPOD, Phosphatome.NET, iEKPD and canonical or historical classification method.

Supplementary Table S3: PubMed IDs scanned during compilation of phosphatase-substrate pairs along with the rationale on why they were included or excluded from the current version.
